# P-1970. Comparative Analysis of Serious Adverse Events Between Azithromycin and Doxycycline: A FAERS-Based Retrospective Study

**DOI:** 10.1093/ofid/ofaf695.2137

**Published:** 2026-01-11

**Authors:** Alvin Sunny, Albin C Sebastian, Linta Susan Kuriakose

**Affiliations:** Square Hospital, West Panthapath, Dhaka, Bangladesh; Square Hospital, West Panthapath, Dhaka, Bangladesh; Square Hospital, West Panthapath, Dhaka, Bangladesh

## Abstract

**Background:**

Azithromycin and doxycycline are among the most frequently prescribed antibiotics for respiratory tract infections, sexually transmitted diseases, and other common conditions. While both are considered generally safe, emerging real-world data suggest differences in their serious adverse event (SAE) profiles. This study aimed to compare the frequency and nature of SAEs associated with azithromycin and doxycycline using the U.S. FDA Adverse Event Reporting System (FAERS) database.

**Figure:**
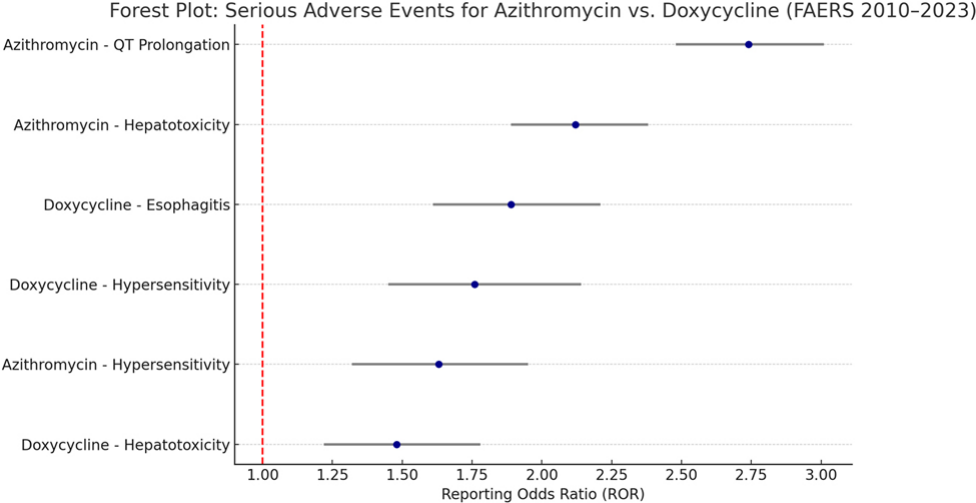
Forest Plot: Serious Adverse Events for Azithromycin vs. Doxycycline (FAERS 2010–2023) This forest plot displays reporting odds ratios (RORs) with 95% confidence intervals for serious adverse events associated with azithromycin and doxycycline based on FAERS data. Azithromycin demonstrated strong associations with QT prolongation (ROR: 2.74) and hepatotoxicity (ROR: 2.12), while doxycycline showed higher signal strength for gastrointestinal events such as esophagitis (ROR: 1.89). Both antibiotics showed overlapping hypersensitivity signals, highlighting the importance of patient-specific risk assessment when prescribing.

**Methods:**

A retrospective pharmacovigilance study was conducted using FAERS data from January 2010 to December 2023. Reports listing azithromycin or doxycycline as primary suspect drugs were extracted. Serious adverse events were defined based on FDA classification (including death, hospitalization, life-threatening events, and disability). Disproportionality analysis was performed using Reporting Odds Ratios (ROR) with 95% confidence intervals (CI). Specific SAE categories examined included cardiac disorders, hepatobiliary disorders, hypersensitivity, and gastrointestinal events.

**Results:**

A total of 38,412 SAE reports were analyzed (azithromycin: 26,754; doxycycline: 11,658). Azithromycin was more frequently associated with cardiac events, particularly QT prolongation and arrhythmias (ROR: 2.74, 95% CI: 2.48–3.01), while doxycycline had higher signal strength for gastrointestinal complications, such as esophagitis and gastritis (ROR: 1.89, 95% CI: 1.61–2.21). Hepatotoxicity signals were notable for both drugs but stronger for azithromycin (ROR: 2.12 vs. 1.48). Both agents had overlapping signals for hypersensitivity reactions, though severe cutaneous reactions were slightly more frequent with doxycycline.

**Conclusion:**

This FAERS-based comparison highlights distinct SAE profiles for azithromycin and doxycycline. Clinicians should consider these differences when selecting empirical antibiotic therapy, especially in patients with pre-existing cardiac or gastrointestinal conditions. Real-world pharmacovigilance data remain crucial for antibiotic risk stratification and safety monitoring beyond controlled trials.

**Disclosures:**

All Authors: No reported disclosures

